# Association of maternal exposure to Superstorm Sandy and maternal cannabis use with development of psychopathology among offspring: the Stress in Pregnancy Study

**DOI:** 10.1192/bjo.2022.595

**Published:** 2023-05-26

**Authors:** Yoko Nomura, Jacob Ham, Patricia M. Pehme, Waiman Wong, Lexi Pritchett, Sima Rabinowitz, Nancy S. Foldi, Veronica J. Hinton, Priya J. Wickramaratne, Yasmin L. Hurd

**Affiliations:** Department of Psychology, CUNY Queens College and Graduate Center, Flushing, New York, USA; and Icahn School of Medicine at Mount Sinai, New York, USA; Icahn School of Medicine at Mount Sinai, New York, USA; Department of Psychology, CUNY Queens College and Graduate Center, Flushing, New York, USA; Department of Psychology, CUNY Queens College and Graduate Center, Flushing, New York, USA; and Department of Radiology, Weill Cornell Medicine, Brain Health Imaging Institute, New York, USA; Columbia University Medical Center and New York State Psychiatric Institute, New York, USA; Icahn School of Medicine at Mount Sinai, New York, USA; and Icahn School of Medicine at Mount Sinai, Addiction Institute of Mount Sinai, New York, USA

**Keywords:** Prenatal stress, natural disaster, perinatal cannabis exposure, developmental psychopathology, longitudinal study

## Abstract

**Background:**

Early-life adverse experiences can elevate the magnitude of the risk of developmental psychopathology, but the potential synergistic effects of multiple factors have not been well studied.

**Aims:**

To determine whether prenatal exposures to maternal stress (Superstorm Sandy) and maternal cannabis use synergistically alter the risk of developmental psychopathology.

**Method:**

The study included 163 children (53.4% girls), longitudinally tracked (ages 2–5 years) in relation to the effects of two early-life adverse exposures (Superstorm Sandy and maternal cannabis use). Offspring were grouped by exposure status (neither, only maternal cannabis use, only Superstorm Sandy or both). DSM-IV disorders for offspring were derived from structured clinical interviews; caregiver-reported ratings of family stress and social support were also assessed.

**Results:**

A total of 40.5% had been exposed to Superstorm Sandy and 24.5% to maternal cannabis use. Offspring exposed to both (*n* = 13, 8.0%), relative to those exposed to neither, had a 31-fold increased risk of disruptive behavioural disorders (DBDs) and a seven-fold increased risk of anxiety disorders. The synergy index demonstrated that offspring with two exposures had synergistic elevation in risk of DBDs (synergy index, 2.06, *P* = 0.03) and anxiety disorders (synergy index, 2.60, *P* = 0.004), compared with the sum of single risks. Offspring with two exposures had the highest parenting stress and lowest social support.

**Conclusions:**

Our findings are consistent with the double-hit model suggesting that offspring with multiple early-life adverse exposures (Superstorm Sandy and maternal cannabis use) have synergistically increased risks of mental health problems. Given the increasing frequency of major natural disasters and cannabis use, especially among women under stress, these findings have significant public health implications.

The developmental origin of health and disease hypothesis postulates that poor gestational quality as measured by exposure to suboptimal conditions, including stress, substance use and maternal infections, has a deleterious impact on development,^1^ particularly affecting the brain. Most neurodevelopmental processes (e.g. neuronal and/or glial genesis, differentiation, migration, synaptogenesis and myelination) are at least initiated, if not completed, during gestation.^2^ These processes are precisely governed by the brain's developmental clock, and any interference during gestation can result in its reprogramming, which can affect the risk for subsequent mental health problems.^3^

An important risk factor for altered neurodevelopmental programming is maternal stress during pregnancy. Preclinical research has demonstrated that maternal stress during gestation adversely affects behavioural and hormonal development.^4^ Although human studies have replicated these findings,^5^ a notable limitation is the ethical introduction of stress in human studies. Consequently, measures of human prenatal stress are generally broad, lack negative valence and typically consider everyday stressful events, which vary greatly among pregnant women. Quasi-experiments based on traumatic major disasters experienced by everyone have provided conditions in which highly acute stress is randomly assigned. Although such disasters have been increasing in occurrence, to date there have been only a few studies examining the impact of natural disasters (the Canadian Ice Storm,^6^ Hurricane Katrina^7^ and Superstorm Sandy^8,9^) or human-made disasters (the Three Mile Island nuclear-plant explosion,^10^ the Chernobyl nuclear plant explosion,^11^ war and terrorism^12,13^) on child health outcomes.

To understand the long-term consequences of early life exposure to stress, a multifactorial conceptualisation of neurodevelopmental and psychiatric disease is emerging, in which multiple biologically significant events (or ‘hits’) are distributed across early life.^14^ One commonly used framework is the double-hit model,^15^ which posits that early life stressors, particularly those occurring during formative developmental periods, may render the organism more vulnerable to other stressors such that the joint effect of the two stressors is greater than the sum of their individual risks. In our current study, we assessed the ‘double-hit’ in children associated with prenatal exposure to stress associated with Superstorm Sandy and perinatal maternal cannabis use. We chose cannabis because the endocannabinoid system is a critical biological system in regulation of neurodevelopmental processes and modulation of stress effects.^16^ Moreover, the legalisation of cannabis and diminishing awareness of its risk is increasing cannabis use among women of reproductive age.^17^ Given recent increases in both major disasters (including the recent COVID-19 pandemic) and cannabis use, examination of the double-hit model could provide considerable public health information regarding healthy child neurobehavioral development.

## Method

This report follows the STROBE reporting guidelines for observational studies.

### Participants

The study included mothers and their children from a longitudinal project (the Stress in Pregnancy (SIP) Study) that investigates the effects of prenatal psychosocial stress, such as that caused by Superstorm Sandy, on offspring development. Superstorm Sandy struck New York City in October 2012, leaving many dead and 8 million households without electricity; there were also gasoline shortages and public transport systems sustained damage.^18^

Pregnant women were initially recruited from two prenatal obstetrics clinics serving a racially and financially diverse population during their second trimester and followed throughout their pregnancy. Exclusion criteria included HIV infection, maternal psychosis, maternal age <15 years, life-threatening maternal medical complications, and congenital or chromosomal abnormalities of the fetus. For details, see Finik and Nomura (2017).^19^ After the birth of the children, child–mother dyads (*N* = 358) were assessed annually. Among these, a subsample of 163 mothers with preschool-aged children (ages 2–5 years; mean (s.d.) = 3.19 (1.17)) were chosen for a diagnostic interview of psychiatric diagnostic outcomes. This subsample of preschool-aged participants did not differ significantly from the rest of the SIP cohort with respect to major demographic characteristics (Supplementary Table 1 available at https://doi.org/10.1192/bjo.2022.595).

All procedures contributing to this work complied with the ethical standards of the national and institutional committees on human experimentation and with the Helsinki Declaration of 1975, as revised in 2008. All procedures involving human subjects and/or patients were approved by the Institutional Review Board (IRB) at the City University of New York. All participants provided written consent according to the protocol approved by the same IRB.

### Measures

#### Exposures

##### Prenatal superstorm sandy

Superstorm Sandy exposure status was defined as whether mothers were pregnant (*N* = 66, or 40.5%) or not (*N* = 97, or 59.5%) during the storm.

##### Perinatal cannabis use

Perinatal cannabis use was assessed during pregnancy and 24 months after the birth of the child. Prenatal cannabis use was ascertained using the Structured Clinical Interview for DSM-IV,^20^ conducted by trained clinical interviewers. Postnatal use was assessed using the Cannabis Use Disorders Identification Test-Revised^21^ at 6, 12, 18 and 24 months and the Mini-International Neuropsychiatric interview at 24 months.^22^ Perinatal cannabis use was coded positive (1) for use prenatally or postnatally (6–24 months). Forty (24.5%) mothers reported perinatal cannabis use.

### Outcomes

#### Primary measure

##### Child psychopathology

The Preschool Age Psychiatric Assessment (PAPA)^23^ was used to ascertain diagnostic outcomes. PAPA is a diagnostic interview of caregivers assessing psychiatric disorders in children between the ages of 2–5 years. It is tailored to measure feelings and behaviours pertinent to young children and enables a definition of age-specific diagnostic criteria when considering developmental changes across the preschool period. The interviews were conducted by clinical interviewers with systematic training and monitoring from the designer of the instrument.^24^ PAPA assesses various DSM-IV diagnostic criteria prevalent in young children, including phobia, anxiety disorders [separation anxiety disorder (SAD), selective mutism, generalised anxiety disorder (GAD), posttraumatic stress disorder (PTSD)], depressive disorders (major depression, dysthymia, and depressive disorders not otherwise specified), and disruptive behavioural disorders (DBDs) [conduct disorder, oppositional defiant disorder (ODD), and attention deficit/hyperactivity disorder (ADHD)]. When answers to gatekeeping questions for each diagnostic category were positive, the interviewers explored symptoms in depth and recorded frequency, duration, and age at first onset, based on a 3 month recall period, as well as lifetime occurrence and diagnoses. Diagnostic outcomes followed the DSM-IV diagnostic algorithms.

PAPA's reliability compares with those of measures widely used for older children and adults.^24^ Interrater reliabilities were fair to good for dysthymia (*k* = 0.72), specific phobia (*k* = 0.46), social phobia (*k* = 0.54), SAD (*k* = 0.60), GAD (*k* = 0.59), selective mutism (*k* = 0.88), conduct disorder (*k* = 0.66), ODD (*k* = 0.62) and ADHD (*k* = 0.78).

#### Secondary measures

##### Postnatal parenting stress

Parenting Stress Index Short-form^25^ is a screening measure for identifying issues related to problems in the parent's or child's characteristics within the family unit. The three subscales capture problems within the family that influence the level of parenting stress. Those three subscales include parental distress (range 12–56), difficult child (range 12–57) and parent–child dysfunctional interaction (range 12–50). The total scale (range 36–138) captures the parenting stress within the family. The α for the total scale is 0.86.

### Social support

MOS-Social Support Survey^26^ measures available social support in four distinct areas (emotional, tangible, affectionate and positive social interaction). The α for the subscales range from 0.91 to 0.96, with a value of 0.97 for the total score. Subscale and total scores range from 1 to 5.

### Potential confounders

#### Child and maternal demographics

Child sex, child race, child ethnicity, maternal age, parents’ marital status, parity, and socioeconomic status (SES) were *a priori* determined as confounders. The child's race and ethnicity were reported by mothers. SES was extracted with four indicators (maternal education, pre-pregnancy occupation prestige,^27^ work and welfare status) using latent class analysis.^28^ For details, see Nomura et al. (2021).^8^

#### Normative prenatal stress

Normative prenatal stress was extracted using latent profile analysis^29^ with the Pregnancy-Related Anxiety Questionnaire-Revised (PRAQ-R),^30^ Perceived Stress Scale (PSS-14),^31^ life events,^32^ maternal depression by the Edinburgh Postnatal Depression Scale (EPDS)^33^ and anxiety by the State-Trait Anxiety Inventory (STAI),^34^ assessed during pregnancy. Internal consistency for the PRAQ-R, PSS-14, EPDS and STAI were α = 0.86, α = 0.91, α = 0.84 and α = 0.89, respectively.

#### Maternal substance use

Maternal substance use during pregnancy was ascertained via face-to-face clinical interview and self-report. Substances reported included tobacco, alcohol, cocaine, heroin, opioids, glue and prescription medications. Positive response was coded as 1, otherwise response was coded as 0.

#### Objective and subjective Superstorm Sandy-related traumas and challenges

Objective Superstorm Sandy-related challenge was assessed with Storm32,^35^ which measures salient aspects of objective disaster exposure. Internal consistency was α = 0.90. Subjective (perceived) post-traumatic stress trauma symptoms were measured by the modified Impact of Events Scale-Revised.^36^ The internal consistency was α = 0.91.

### Statistical method

Children were grouped by maternal exposure status to Superstorm Sandy and cannabis. The exposure-group variable had four categories: children exposed to neither Superstorm Sandy nor cannabis (reference), Superstorm Sandy only, cannabis only, and both Superstorm Sandy and cannabis.

Cumulative lifetime risk of various psychiatric disorders was estimated using survival analysis techniques by means of a modified Kaplan–Meier method.^37^ Prior to the analysis, the proportional hazard assumption was tested with each diagnostic outcome, using the Schoenfeld residuals.^38^ Then, equality of survival distributions for the four groups was estimated using log-rank test. A Cox proportional hazards regression model^39^ was then used in three stages. The model in step 1 included sociodemographic and psychosocial confounders. The model in step 2 included postnatal psychosocial environment scales (family stress and social support) to evaluate the unique contribution of postnatal psychosocial environment over and above demographic and psychosocial confounders in step 1. In step 3, only the exposure group variable was included to evaluate the unique contribution of our exposures over and above contributions of the demographic confounders and postnatal psychosocial environment.

Lastly, to estimate the hazard risks of each diagnostic outcome, Cox proportional hazards regression models were then used in the three exposed groups relative to the reference group. Additive interaction – defined as the departure of disease rates from an additive model,^40,41^ based on Rothman's ‘index of synergism’ – was chosen to test the double-hit model. A synergy index was calculated to examine the magnitude of additive interaction. Synergy index values of 1 indicated that the sum of the two risks (Superstorm Sandy and cannabis use) equalled the risk of exposure to both, whereas synergy index values greater than 2 indicated appreciable synergistic acceleration of risk.^40^ To assess the significance of the synergy index, standard errors of each synergy index were calculated based on the approach of Cortina-Borja and colleagues.^42^ A 4 × 2 table of the numbers of cases and controls (of each disorder) in four groups by the two risks (Superstorm Sandy and cannabis use) was constructed. Then, with *n*_1_, *n*_2_ …. *n*_8_ as the values of the eight cells, application of the delta method^43^ yielded an asymptotic normal approximation to the standard error of ln(SI) as: 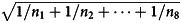, where SI is the synergy index. The statistic *Z* = ln(SI)/s.e.(ln(SI)) had an asymptotically standard normal distribution under the null hypothesis of no interaction. We obtained *P*-values based on this method.

We then estimated the differences in levels of psychosocial family environment (family stress and social support) from 2–5 years of age. Generalised estimating equations (GEE)^44^ facilitated analysis of longitudinal designs^45^ by nesting multiple assessments at different ages and evaluating the overall differences among the four groups with all covariates. To explore group differences, pairwise comparisons were assessed *a priori* without adjustment of multiple testing.

The *a priori* level of significance, based on two-sided tests, was set at *P* = 0.05. All analyses were conducted using IBM SPSS Statistics, version 28.

## Results

### Demographic, psychosocial, and lifestyle characteristics

Table 1 shows the overall characteristics of the 163 participants as a whole and by exposure group; 53% were girls. Participants were racially diverse. In racial categories, 31 (19.0%) were Black, 15 (9.2%) were Asian and 50 (30.7%) were mixed or other non-White races. Ninety-four (57.7%) were Hispanic and 69 (42.3%) were non-Hispanic. There were no major differences among the four exposure groups with respect to major demographic variables. However, psychosocial and behavioural characteristics including cigarette smoking were more prevalent and normative prenatal stress (*P* = 0.01) was higher in the two cannabis exposure groups (cannabis-only and both Superstorm Sandy and cannabis).

**Table 1:** Demographic characteristics of the total sample population and subgroups

		Exposure groups				
	Total sample (*n* = 163)	Reference (*n* = 70)	CB only (*n* = 27)	SS only (*n* = 53)	Both (*n* = 13)	Statistics
Child race, *N* (%)						
White	67 (41.1)	25 (35.7)	10 (37.0)	27 (50.9)	5 (38.5)	
Black	31 (19.0)	14 (20.0)	9 (33.3)	6 (11.3)	2 (15.4)	
Asian	15 (9.2)	11 (15.7)	0	4 (7.5)	0	
Mixed/other	50 (30.7)	20 (28.6)	8 (29.6)	16 (30.2)	6 (46.2)	χ^2^(9) = 14.75, *P* = 0.10
Child ethnicity, *N* (%)						
Hispanic	94 (57.7)	39 (55.7)	12 (44.4)	33 (62.3)	10 (76.9)	
Non-Hispanic	69 (42.3)	31 (44.3)	15 (55.6)	20 (37.7)	3 (23.1)	χ^2^(3) = 4.48, *P* = 0.21
Child sex, *N* (%)						
Girls	87 (53.4)	30 (42.9)	17 (63.0)	31 (58.5)	9 (69.2)	
Boys	76 (46.6)	40 (57.1)	10 (37.0)	22 (41.5)	4 (30.8)	χ^2^(3) = 5.98, *P* = 0.11
Maternal age (years), mean (s.d.), range	27.80 (5.92), 17-43	27.82 (6.06)	27.15 (5.86)	28.83 (6.09)	24.88 (3.51)	F(3,159) = 1.72. *P* = 0.17
Paternal age (years)^a^, mean (s.d.), range	30.37 (6.94), 17–53	31.37 (7.22)	27.58 (5.71)	31.00 (7.01)	28.15 (5.93)	F(3, 154) = 2.54, *P* = 0.06
Parity, mean (s.d.), range	2.08 (1.60), 0–7	2.04 (1.62)	1.81 (1.57)	2.11 (1.63)	2.69 (1.38)	F(3,159) = 0.90, *P* = 0.44
Maternal marital status, *N* (%)						
Married	79 (48.5)	38 (54.3)	8 (29.6)	28 (52.8)	5 (38.5)	
Common law marriage	8 (4.9)	3 (4.3)	1 (3.7)	2 (3.8)	2 (15.4)	
Single	70 (42.9)	28 (40.0)	17 (63.0)	19 (35.8)	6 (46.2)	
Separated/divorced	6 (3.7)	1 (1.4)	1 (3.7)	4 (7.5)	0	χ^2^(9) = 13.0, *P* = 0.16
Maternal socioeconomic status, *N* (%)						
High	37 (22.6)	17 (23.9)	4 (14.8)	15 (28.8)	1 (7.7)	
Medium	69 (42.1)	30 (42.3)	13 (48.1)	18 (34.6)	8 (61.5)	
Low	58 (35.4)	24 (33.8)	10 (37.0)	19 (36.5)	4 (30.8)	χ^2^(6) = 5.69, *P* = 0.46
Maternal prenatal stress, *N* (%)						
High	30 (42.9)	30 (42.9)	2 (7.4)	22 (41.5)	2 (15.4)	
Medium	31 (44.3)	31 (44.3)	16 (59.3)	24 (45.3)	8 (61.5)	
Low	9 (12.9)	9 (12.9)	9 (33.3)	2 (13.2)	3 (23.1)	χ^2^(6) = 16.9, *P* = 0.01
Maternal tobacco use during pregnancy, *N* (%)	14 (8.6)	1 (1.4)	8 (29.6)	1 (1.9)	4 (30.8)	χ^2^(3) = 39.97, *P* < 0.0001
Maternal alcohol use during Pregnancy, *N* (%)	10 (6.1)	4 (5.7)	1 (3.7)	3 (5.7)	2 (15.4)	χ^2^(3) = 2.25, *P* = 0.52
Maternal PTSD symptoms, mean (s.d.), range	7.34 (11.64), 0–88	4.60 (6.92)	14.86 (20.73)	7.12 (9.70)	7.44 (6.52)	F(3,159) = 5.49, *P* = 0.001
Maternal severity of SS stress, mean (s.d.), range	2.87 (2.67), 0–15	2.22 (1.86)	3.37 (3.27)	3.94 (3.01)	3.61 (3.01)	F(3,159) = 2.49, *P* = 0.06
Birthweight (kg), mean (s.d.), range	3.22 (0.54), 1.49–4.93	3.18 (0.45)	3.06 (0.47)	3.33 (0.55)	3.28 (0.92)	F(3,159) = 1.77, *P* = 0.15
Gestational age at delivery (weeks), mean (s.d.), range	38.79 (1.94), 32.0–42.0	38.49 (2.03)	38.39 (2.20)	39.32 (1.62)	38.92 (1.68)	F(3,159) = 2.55, *P* = 0.06

a. There were five cases with missing information. Reference group: no exposure to cannabis (CB) or Sandy Storm (SS); CB only: exposed to only cannabis; SS only: exposed to only SS; both: exposed to both cannabis and SS.

### Proportional hazards assumption and differential risk of psychopathology by Superstorm Sandy and cannabis exposures

**Fig. 1:**
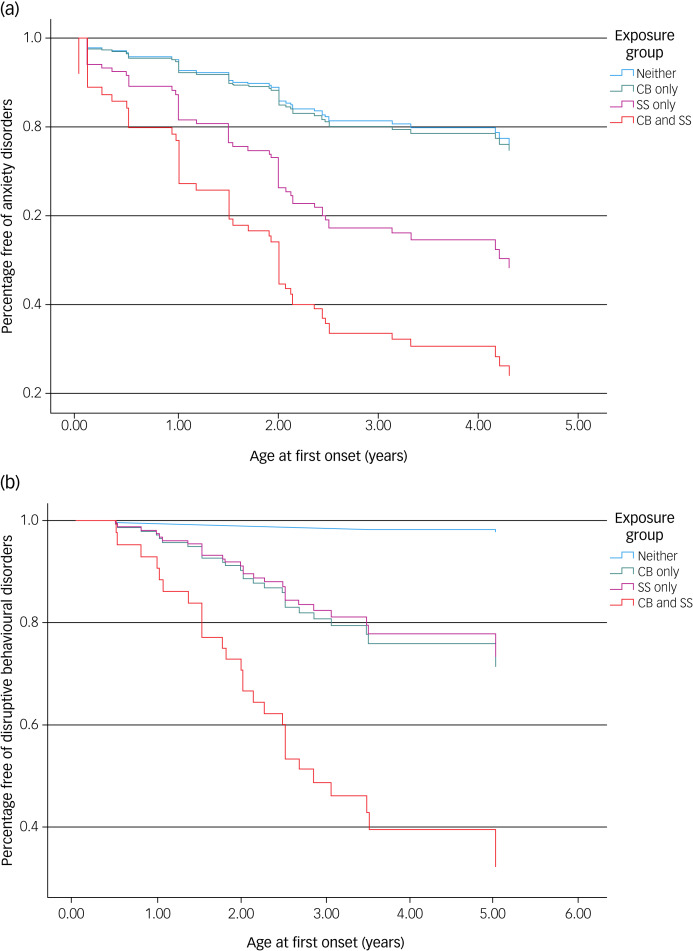
Survival curves for any anxiety disorder and any disruptive behavioural disorder among children exposed to Superstorm Sandy (SS) and/or maternal cannabis (CB) use. Blue line, children not exposed to either SS or CB (*N* = 70); green line, children exposed to only CB (*N* = 27); red line, children exposed to only SS (*N* = 53); orange line, children exposed to both (*N* = 13). Significant difference among groups was found in the test of equality of strata (log-rank test), χ^2^(3) = 22.22, *P* < 0.0001 for any anxiety disorder and χ^2^(3) = 26.84, *P* < 0.0001 for any disruptive disorder. The cumulative risks of lifetime disorders were estimated after adjusting for confounders.

Inspection of the Schoenfeld residuals by our predictor on functions of time showed that there was no violation in the assumption with any of the diagnostic outcomes. Figure 1 shows the patterns of onset for any anxiety disorder (a) and any DBD (b) among children in the four groups. The test of equality of strata showed significant difference in the patterns of onset in any anxiety disorder (χ^2^(3) = 22.22, *P* < 0.0001) and any DBD (χ^2^(3) = 26.84, *P* < 0.0001). Among individual disorders, significant differences were found for SAD (*P* = 0.001), GAD (*P* < 0.0001), social phobia (*P* < 0.0001), dysthymia (*P* = 0.002), conduct disorder (*P* < 0.0001), ODD (*P* < 0.0001) and ADHD (*P* < 0.0001).

Table 2 shows the unique contribution of postnatal psychosocial factors (step 2) and exposure group (step 3) in diagnostic outcomes. With the exception of any depressive disorders, postnatal psychosocial environment on the composite disorder outcomes, including any anxiety (Δχ^2^(2) = 6.6, *P* = 0.04), any phobia (Δχ^2^(2) = 8.7, *P* = 0.01) and any DBD (Δχ^2^(2) = 7.1, *P* = 0.03), had significant contribution to the elevated cumulative risks over and above the confounders’ effects (column 3). Furthermore, our predictor (exposure group) was uniquely associated with elevated risks for any anxiety disorder (Δχ^2^(3) = 22.9, *P* < 0.001), any depressive disorder (Δχ^2^(2) = 12.1, *P* = 0.007) and any DBD (Δχ^2^(3) = 16.6, < 0.001) over and above what was already explained in steps 1 and 2 (column 4).

**Table 2:** The effect of exposures to maternal Superstorm Sandy (SS) and cannabis use (CB) in consideration of multiple potential confounders on child diagnostic outcomes

Outcomes	Step 1^a^ (covariate only)		Step 2^b^ (postnatal environment)		Step 3^c^ (exposure)		Overall model	
	χ^2^(11)	*P*	Δχ^2^(2)	*P*	Δχ^2^(3)	*P*	χ^2^(16)	*P*
**Any anxiety disorder**	12.22	0.35	6.60	0.04	22.94	<0.001	41.75	<0.001
Separation anxiety disorder	10.26	0.51	5.06	0.08	13.57	0.004	30.29	0.02
Generalised anxiety disorder	22.14	0.02	1.54	0.46	19.04	<0.001	60.73	<0.001
**Any phobia**	18.39	0.07	8.70	0.01	2.64	0.45	29.22	0.02
Specific phobia	20.28	0.04	10.33	0.006	1.71	0.63	30.97	0.01
Social phobia	17.73	0.09	2.74	0.25	19.15	<0.001	56.30	<0.001
**Any depressive disorder**	10.25	0.51	1.17	0.56	12.10	0.007	26.28	0.05
Dysthymia	10.25	0.51	1.17	0.56	12.10	0.007	26.28	0.05
**Any disruptive behavioural disorder**	10.31	0.51	7.07	0.03	16.63	<0.001	36.25	0.003
Conduct disorder	7.76	0.74	3.86	0.15	10.60	0.01	25.99	0.05
Oppositional defiant disorder	14.88	0.19	6.83	0.03	14.20	0.003	36.72	0.002
Attention deficit hyperactivity disorder	6.27	0.86	7.76	0.02	18.42	<0.001	41.21	<0.001

Log-rank test was used to estimate the equality of survival distributions among the four groups in survival analysis by means of a modified Kaplan–Meier method.

a. Step 1 includes all of the 11 covariates including child race and ethnicity, child sex, maternal age, marital status of the parents, parity, socioeconomic status, prenatal normative stress, prenatal substance use, post-traumatic stress symptoms related to SS and objective SS stress. χ^2^(11) represents the overall model fit of the covariates-only model

b. Step 2 includes an additional two indicators of postnatal environment (postnatal family stress and social support). Δχ^2^(2) shows the changes in the model fit from the covariates-only model (step 1).

c. Step 3 includes the exposure variable with four groups (neither, only CB, only SS, both CB and SS). Δχ^2^(3) shows the changes in the model fit from the previous (step 2).

Rates of individual disorders are shown in the first section of the column (cumulative rates over 5 years) in Table 3. For anxiety disorders, children exposed to both Superstorm Sandy and cannabis use had the highest rates of SAD (69.2%), GAD (46.2%) and selective mutism (7.7%), and over 75% had one form of anxiety disorder. Superstorm Sandy-only children had the next highest prevalence of anxiety disorders (37.7% SAD, 13.2% GAD and 47.2% any anxiety disorder), followed by cannabis-only children (18.5% SAD, 7.4% GAD and 25.9% any anxiety disorder). Within phobias, over 60% of children exposed to both and 18.5% in the cannabis-only group had social phobia. For DBDs, children exposed to both had the highest rates of any DBD (30.8%) – conduct disorder (15.4%), ODD (53.8%) and ADHD (46.2%) – followed by the two single exposure groups. The reference group, children exposed to neither, had the lowest rates of any DBD (2.9%) – conduct disorder (1.4%), ODD (2.9%) and ADHD (2.9%). Approximately one-quarter of children exposed to both Superstorm Sandy and cannabis use had dysthymia (23.1%).

**Table 3:** Risk of disorders among children with maternal cannabis (CB) and Superstorm Sandy (SS) exposure

	Cumulative rates over 5 years (%)				Hazard ratio (HR)							Synergy index (SI)	
	Reference (*n* = 70)	CB only (*n* = 27)	SS only (*n* = 53)	Both (*n* = 13)	Reference	CB only HR (95% CI)	*P*-value	SS only HR (95% CI)	*P*-value	Both HR (95% CI)	*P*-value	SI, *z*-score	*P-*value
**Any anxiety**^a^	20.0	25.9	47.2	76.2	1.0	1.35 (0.38–4.05)	.72	3.30 (2.22–12.62)	0.0002	7.89 (2.53–24.41)	0.0004	2.60, 3.55	0.004
SAD	18.6	18.5	37.7	69.2	1.0	1.14 (0.32–4.01)	.89	3.13 (1.25–7.87)	0.02	6.59 (1.96–22.28)	0.002	2.90, 5.81	<0.001
GAD	1.4	7.4	13.2	46.2	1.0	7.54 (0.48–119.62)	.15	9.49 (1.00–91.65)	0.05	48.58 (4.96–475.65)	0.0005	3.17, 2.76	0.006
PTSD	0	0	3.8	0	1.0	–		–		–		–	
\Selective mutism	1.4	3.7	3.8	7.7	1.0	–		–		–		–	
**Any phobia**^a^	38.6	51.9	41.5	69.2	1.0	1.30 (0.60–2.79)	0.51	1.44 (0.74–2.80)	0.29	2.18 (0.84–5.65)	0.10	1.59, 1.62	0.105
Specific phobia	34.3	51.9	37.7	46.2	1.0	1.52 (0.68–3.36)	0.31	1.62 (0.81–3.26)	0.18	1.46 (0.48–4.67)	0.51	0.40, 0.04	0.968
Social phobia	8.6	18.5	9.4	61.5	1.0	1.62 (0.37–7.18)	0.52	0.94 (0.24–3.60)	0.92	17.83 (3.80–83.66)	0.0003	6.57, 6.10	<0.001
Agoraphobia	2.9	0	0	0	1.0	–		–		–		–	
**Any DBD**	2.9	14.8	11.3	30.8	1.0	9.41 (1.64–53.99)	0.01	6.40 (1.26–22.55)	0.03	31.19 (4.97–195.90)	0.0002	2.06, 2.15	0.032
CD	1.4	11.1	7.5	15.4	1.0	5.86 (0.79–43.46)	0.08	2.78 (0.44–17.58)	0.27	15.44 (2.33–102.37)	0.005	2.32, 2.10	0.036
ODD	2.9	14.8	13.2	53.8	1.0	6.86 (1.00–48.12)	0.05	3.38 (0.57–20.26)	0.18	16.24 (2.48–106.20)	0.004	1.98, 1.96	0.049
ADHD	2.9	11.1	11.3	46.2	1.0	2.09 (0.41–10.80)	0.06	1.81 (0.45–7.23)	0.40	26.27 (4.53–152.41)	0.0003	13.30, 6.20	<0.001
**Any depression**	1.4	0	5.7	23.1	1.0	–		3.83 (0.16–89.94)	0.40	54.73 (1.18–300.73)	0.03	–	
MDD	0	0	0	0	1.0	–		–		–			
Dysthymia	1.4	0	5.7	23.1	1.0	–		3.83 (0.16–89.94)	0.40	54.73 (1.18–300.73)	0.03	–	
DDNOS	0	0	0	0	1.0	–		–		–		–	

a. Anxiety disorders do not include phobia and phobia does not include anxiety disorders.

RR = relative risk; SAD = separation anxiety disorder; GAD = generalised anxiety disorder; PTSD = post-traumatic stress disorder; MDD = major depressive disorder; DDNOS = depressive disorder – not otherwise specified; DBD = disruptive behavioural disorder; CD = conduct disorder; ODD = oppositional defiant disorder; ADHD = attention-deficit hyperactivity disorder.

Analysis was based on the proportional hazard model with covariates including child sex, child race, child ethnicity, maternal age, marital status of the parents, parity, family socioeconomic status, prenatal substance use, prenatal stress, post-traumatic stress symptoms due to Superstorm Sandy and severity of objective Superstorm Sandy exposures, as well as postnatal family stress and social support at age 3.

SI was calculated as follows. S_AB_ = (hR_AB_−1)/(HR_A_ + HR_B_−2); ln (S_AB_) = sqrt [Σ*n_ij_* ], where *i* is a subsample of each group for positive and *j* is for negative disorders.

Lifetime cumulative risks for each disorder were estimated for the three exposure groups relative to the reference group. These risks are shown in the second column of Table 3. A notable finding was the risk of DBDs for children exposed to both. These children had an over 31-fold increased risk for any DBD [hazard ratio (HR) = 31.2, *P* = 0.0002]. Among the specific DBDs, those exposed to both had a 26-fold increased risk for ADHD (HR = 26.3, *P* = 0.0003), a 16-fold increased risk for ODD (HR = 16.2, *P* = 0.004) and a 15-fold increased risk for conduct disorder (HR = 15.4, *P* = 0.005). Children exposed to both had a 54-fold increased risk for any depressive disorder (HR = 54.7, *P* = 0.03) and a more than seven-fold increased risk for any anxiety disorder (HR = 7.89, *P* = 0.0004). Among specific anxiety and depressive disorders, those exposed to both risks had a >54-fold increased risk for dysthymia (HR = 54.7, *P* = 0.03), a >48-fold increased risk for GAD (HR = 48.6, *P* = 0.0005) a >17-fold increased risk for social phobia (HR = 17.8, *P* = 0.0003) and a six-fold increased risk for SAD (HR = 6.6, *P* = 0.002). Children exposed to Superstorm Sandy only also had a substantial increased risk for any DBD (HR = 6.4, *P* = 0.03) and any anxiety disorder (HR = 3.3, *P* = 0.0002), whereas children exposed to cannabis only had an increased risk for any DBD (HR = 9.4, *P* = 0.01).

The synergy index for magnitude of additive interaction is shown in the third section of Table 3. Among overall categories, any DBD (synergy index, 2.06, *P* = 0.03) and any anxiety disorder (synergy index, 2.60, *P* = 0.004) showed significant synergistically increased risk. Among individual risks, risks for ADHD (synergy index, 13.30, *P* < 0.001), social phobia (synergy index, 6.57, *P* < 0.001), GAD (synergy index, 3.17, *P* = 0.006), SAD (synergy index, 2.90, *P* < 0.001), conduct disorder (synergy index, 2.32, *P* = 0.04) and ODD (synergy index, 1.98, *P* = 0.05) among children exposed to both were synergistically elevated.

### Characteristics of postnatal family environment by exposure groups

Table 4 shows overall group differences in parenting stress and social support, and pairwise comparisons (last column) among the four groups. Generally, children exposed to both Superstorm Sandy and cannabis use had the highest levels of parenting stress and lowest levels of social support, followed by the cannabis-only group. Specifically, total parenting stress was highest in the group exposed to both. Available social support in all areas (emotional support, tangible support, affectionate support and positive social interaction) was lowest among the group exposed to both. However, families that experienced Superstorm Sandy only had comparable scores to the reference group that experienced neither Superstorm Sandy nor cannabis use.

**Table 4:** Characteristics of postnatal family environment in relation to maternal cannabis use and/or Superstorm Sandy exposure

	Reference [group 1], (*n* = 70), Mean (s.e.)	CB Only [group 2], (*n* = 27), Mean (s.e.)	SS Only [group 3], (*n* = 53), Mean (s.e.)	Both [group 4], (*n* = 13), Mean (s.e.)	Overall difference	Significant group^a^ differences
**Parenting stress**						
Difficult child	23.80 (0.84)	26.80 (1.66)	23.49 (1.01)	29.93 (2.23)	χ^2^(3) = 10.13, *P* = 0.02	1,3 < 4
Parental distress	25.55 (1.02)	30.40 (1.82)	26.42 (1.20)	31.22 (1.92)	χ^2^(3) = 8.80, *P* = 0.03	1 < 2,4
Parent–child dysfunctional interaction	19.50 (0.72)	19.15 (1.27)	17.75 (0.64)	20.59 (1.43)	χ^2^(3) = 5.58, *P* = 0.13	3 < 4
*Total parenting stress*	67.95 (2.05)	75.88 (3.61)	67.19 (2.34)	80.35 (4.16)	χ^2^(3) = 10.70, *P* = 0.01	1,3 < 4
**Social support**						
Emotional support	4.21 (0.11)	3.70 (0.19)	4.01 (0.14)	3.30 (0.23)	χ^2^(3) = 14.23, *P* = 0.003	1 > 2,4; 2,3 > 4
Tangible support	3.94 (0.12)	3.51 (0.18)	3.84 (0.15)	3.56 (0.16)	χ^2^(3) = 5.00, *P* = 0.17	None
Affectionate support	4.29 (0.10)	3.92 (0.17)	4.21 (0.13)	3.81 (0.18)	χ^2^(3) = 6.24, *P* = 0.10	1 > 4
Positive social interaction	4.17 (0.12)	3.76 (0.19)	4.12 (0.14)	3.54 (0.18)	χ^2^(3) = 9.58, *P* = 0.02	1,3 > 4
*Total social support*	4.15 (0.11)	3.70 (0.17)	4.02 (0.13)	3.48 (0.17)	χ^2^(3) = 11.90, *P* = 0.008	1 > 2,3 > 4

a. The number refers to the group membership: group 1 (reference), exposed to neither cannabis (CB) nor Sandy Storm (SS); group 2 (CB only), exposed to only cannabis; group 3 (SS only), exposed to only SS; group 4 (both), exposed to both CB and SS. Only significant results from the pairwise comparisons are reported.

Analysis was based on the proportional hazard model with covariates including child sex, child race, child ethnicity, maternal age, marital status of the parents, parity, family socioeconomic status, prenatal substance use, prenatal stress, posttraumatic stress symptoms due to Superstorm Sandy and severity of objective Superstorm Sandy exposures.

## Discussion

This investigation builds on our longitudinal study SIP study, which follows a cohort of mothers and children born around the time of Superstorm Sandy. To our knowledge, this is the first study to examine the joint effects of prenatal exposure to a major natural disaster and early life exposure to the mother's cannabis use on psychiatric outcomes among preschool-aged children. The results show that whereas both Superstorm Sandy and maternal cannabis use alone were associated with increased risks of DBDs and anxiety disorders, the synergistic risk of joint exposure was appreciably greater than the sum of the two individual risks, specifically for SAD, GAD, depressive disorders, social phobia, conduct disorder, ODD and ADHD. Our findings are consistent with the double-hit model of a synergistic impact of early life stress with cannabis exposure on future psychopathology risk.^15^

Our findings of heightened risks for developmental psychopathologies in children of mothers who experienced prenatal stress and perinatal cannabis use are in line with preclinical studies demonstrating that these prenatal environmental exposures can induce neurobehavioral disturbances relevant to psychopathology risk in offspring.^3^ Moreover, early life stress is known to affect the endocannabinoid system, which has been well documented to regulate biological stress processes. This strong interaction between stress and endocannabinoid systems, in turn, influences neural networks underlying the regulation of emotions.^46^ The fact that the combination of stress and cannabis exposure resulted in a synergistically elevated risk for psychopathology has important public health and policy implications. However, as multiple factors need to be considered regarding the current population, caution must be used in interpreting the associations between cannabis, in addition to Superstorm Sandy, on child outcomes only in the framework of a double-hit model. Nevertheless, the results of this study provide an important foundation for future studies to replicate and expand aspects of causality.

Our study had several strengths. First, unlike most studies that define prenatal stress as everyday stress, maternal psychopathology or low SES, our study examined the effects of a large-scale natural disaster on developmental programming. Second, with the additional measurement of perinatal maternal cannabis use, we were able to obtain evidence of a synergistic, double-hit impact of both stressors on the risks of developmental psychopathology on offspring. Third, the use of a structured interview for child psychopathology instead of a caregiver-report scale, provided greater confidence in our estimates of increased risks for developmental psychopathology. Fourth, there are strengths in the use of longitudinal statistics: survival analysis enabled us to estimate the cumulative risk of each disorder over the first 5 years of life; and GEE enabled us to leverage the parallel measure of postnatal psychosocial family environment assessed annually from 2–5 years of age. GEE evaluate differences in postnatal stress and social support while incorporating missing data and intra-correlations within the individual participants. Fifth, the study provided a real-life model of the significant consequences of altered neurodevelopmental programming.

Our study also had limitations. It was based on a sample of 163 children, which was a subsample of a larger cohort (*N* = 358), as only children of preschool age were given the structural clinical interviews. However, as Supplementary Table 1 shows, there were no notable differences in demographic and stress variables between the subsample and the rest of the sample. Second, although clinical interviews are the gold standard for measuring preschool psychopathology, there is still the risk of shared variance due to having the same caregiver provide all reports. It is also known that maternal depression can influence mothers’ ratings of their children. We attempted to mitigate this risk by controlling for normative stress extracted by the latent profile analysis including maternal depression, anxiety and perceived stress. Third, although the data indicated increased synergistic risk for psychopathology in young children exposed to both Superstorm Sandy and cannabis use, the sample size of this highest risk group was small (*n* = 13), and it is possible that the small sample size may have inflated the risk estimates, leading to a lack of generalisability. However, it is important to note that we examined the cumulative risk over time (i.e., HR). HRs provide more accurate and robust estimates than odds ratios when estimates are based on a small sample size. Nevertheless, readers should interpret the magnitude of risks, especially for ADHD, conduct disorder and ODD, with caution. Fourth, there were no psychiatric diagnoses of mothers and fathers, which may have genetic contributions to offspring's diagnostic outcomes. Fifth, although exposure timing during pregnancy is an important developmental factor, our relatively small sample size (*n* = 163) prohibited further division by trimester of the exposed group. Finally, although our results show a robust and alarming increased risk associated with exposure to both Superstorm Sandy and cannabis use over and above various potential confounders and the postnatal psychosocial family environment in the staged analysis, it is still possible that other important confounders were overlooked. For example, perinatal cannabis exposure could be associated with the mother's prior abuse history.^47^ Moreover, potential neurotoxicity associated with early-life exposure to cannabis was not examined in the offspring.

The current findings have significant public health implications, given the increasing frequency of major disasters (e.g., the COVID-19 pandemic, wildfires and extreme tropical storms) and the increase in legalisation and use of cannabis, especially among women under stress.^46^ It is critical not only to acknowledge and tend to the needs of pregnant women given the enormous potential impact of stress from such disasters but also to educate and caution around the use of cannabis, be it recreational or to cope with life stressors.

## Data Availability

The data that support the findings of this study are available from the corresponding author (Y.N.) upon reasonable request.

## Supplementary material

For supplementary material accompanying this paper visit http://doi.org/10.1192/bjo.2022.595. click here to view supplementary material
