# Supplementary material for: Association of maternal exposure to Superstorm Sandy and maternal cannabis use with development of psychopathology among offspring: the Stress in Pregnancy Study
Source: BJPsych Open. 2023 May 26;9(3):e94. doi: 10.1192/bjo.2022.595 (PMC10228222; doi:10.1192/bjo.2022.595)
Supplement: Supplementary file 1 [file S2056472422005956sup001.docx]

**Supplementary Table 1** Characteristics of participants in the Stress-In-Pregnancy project that were included versus those not included in the study.

|  | Included  (n=163) | Not included  (n=195) | Statistics, p-value |
| --- | --- | --- | --- |
| **Maternal age**, Mean (SD) | 27.80 (5.92) | 28.50 (6.36) | F=2.40, p=.12 |
| **Paternal age**, Mean (SD) | 30.37 (6.94) | 31.17 (7.83) | F=0.64, p=.42 |
| **Parity**, Mean (SD) | 2.87 (2.67) | 2.62 (1.60) | F=3.29, p=.07 |
| **Maternal anxiety ^a^**, Mean (SD) | 77.21 (20.48) | 76.33 (20.64) | F=0.15, p=.70 |
| **Maternal depression ^b^,** Mean (SD) | 9.33 (4.91) | 9.27 (4.68) | F=0.05, p=.83 |
| **Objective Sandy stress ^c^**, Mean (SD) | 2.87 (2.81) | 2.94 (2.89) | F=0.13, p=.72 |
| **Race^d^**, N (%)  White  Black  Hispanic  Asians  Others | 33 (20.2)  27 (16.6)  75 (46.0)  16 (9.8)  12 (7.4) | 38 (19.5)  45 (23.1)  91 (46.7)  15 (7.7)  6 (3.1) | X^2^(4)=5.61, p=.23 |
| **Child Sex**, N (%)  Male  Female | 76 (46.6)  87 (53.4) | 106 (54.4)  89 (45.6) | X^2^(1)=2.12, p=.15 |
| **Marital status**, N (%)  Married  Common Law  Single  Widowed  Divorced/Separated | 79 (48.5)  8 (4.9)  70 (42.9)  0  6 (3.7) | 84 (43.1)  7 (3.6)  100 (51.3)  1 (0.5)  3 (1.5) | X^2^(4)=3.94, p=.42 |
| **Maternal Education**, N (%)  Elementary School  Some high school  High School/GAE  Some College  Associate Degree (2-year college)  Bachelor’s degree (4-year college)  Graduate/professional | 4 (2.5)  11 (6.8)  30 (18.6)  43 (26.7)  19 (11.8)  32 (19.9)  22 (13.7) | 5 (2.6)  29 (14.9)  42 (21.6)  44 (22.7)  14 (7.2)  30 (15.5)  30 (15.5) | X^2^(6)=9.29, p=.16 |
| **Tobacco use in pregnancy ^d^**, N (%) | 17 (10.6) | 19 (9.9) | X^2^(1)=0.05, p=.82 |
| **Alcohol use in pregnancy ^d^**, N (%) | 12 (7.6) | 13 (6.8) | X^2^(1)=0.09, p=.77 |

**^a^** Based on the State-Trait Anxiety Inventory (Spielberger, 1989)

**^b^** Based on the Edinburgh Postnatal Depression Scale (Murray & Carothers, 1990)

**^c^** Based on Storm32 (Yong, et al, 2015)

**^d^** Based on the self-report at the entry of the study during pregnancy
